# Teenage childbearing and school dropout in a sample of 18,791 single mothers in Cameroon

**DOI:** 10.1186/s12978-021-01323-4

**Published:** 2022-01-15

**Authors:** Joëlle L. Sobngwi-Tambekou, Marthe Tsague-Agnoux, Léopold K. Fezeu, Flavien Ndonko

**Affiliations:** 1 RSD Institute, Rue de l’Université, Po Box 7535, Yaoundé, Cameroon; 2 Université Catholique d’Afrique Centrale (UCAC), Yaoundé, Cameroon; 3 London School of Economics and Political Science, London, UK; 4 Sorbonne Paris Nord University, Inserm, Inrae, Cnam, Nutritional Epidemiology Research Team (EREN), Epidemiology and Statistics Research Center-University of Paris (CRESS), 93017 Bobigny, France; 5 Deutsche Gesellschaft für Internationale Zusammenarbeit (GIZ) GmbH, Yaoundé, Cameroon

**Keywords:** Teenage pregnancy, Single mothers, School dropout, Family support, School continuity, Grossesse adolescente, Mères célibataires, Abandon scolaire, Soutien familial, Continuité scolaire

## Abstract

**Background:**

Adolescent childbearing increases the risk of adverse health and social consequences including school dropout (SDO). However, it remains unclear why some teenage mothers drop out of school and others do not, especially in sub-Saharan Africa settings. We aimed to investigate the background and behavioral characteristics of single mothers, associated with school dropout in a sample of 18,791 Cameroonian girls, who had their first child during adolescence.

**Methods:**

We used data from a national registry of single mothers, collected during the years 2005–2008 and 2010–2011. Both bivariate analysis and logistic binary regression models were used to explore the relationship between adolescence motherhood and SDO controlling for a range of socio-economic, family, sexual and health seeking behavior characteristics.

**Results:**

Among the 18,791 single mothers, 41.6% had dropped out of school because of pregnancy. The multivariable regression model showed that SDO was more common in those who were evicted from their parental home (aOR: 1.85; 95% CI: 1.69–2.04), those who declared having other single mothers in their family (aOR: 1.16; 95% CI 1.08–1.25) and in mothers who had their first child before 15. Using modern contraceptive methods, having declared no sexual partner during the last year and having less than 2 children were associated with a reduced likelihood of school dropout.

**Conclusions:**

Strong social support is essential to ensure school continuity in this vulnerable population. Dropping out of school may put the teenage mother more at risk of unsafe health behaviour and new pregnancies.

## Plain language summary

There is compelling, worldwide evidence that pregnancy and birth during teenage years are significant contributors to high school dropout rates, especially in developing countries. Research has also shown that education continuity of teenage mothers can lessen the long term negative social consequences of teenage pregnancies and childbearing. In this study, we investigated the factors associated with school dropout in a sample of Cameroonian teenage mothers.

The information collected included socio-economic, family, sexual characteristics, and health-seeking behavior.

Among the 18,791 single mothers, 41.6% had dropped out of school because of pregnancy. School dropout was more common in those who were evicted from their parental home, those who declared having other single mothers in their family and in mothers who had their 1st child before 15. Using modern contraceptive methods, having declared no sexual partner during the last year and having less than 2 children were associated with a reduced likelihood of school dropout.

In conclusion, strong social support is essential to ensure school continuity in this vulnerable population. Dropping out of school may put the teenage mother more at risk of unsafe health behaviour and new pregnancies.

## Résumé

**Contexte:**

La maternité chez les adolescentes augmente le risque d’évènements sanitaires et sociaux néfastes, notamment l'abandon scolaire (SDO). Cependant, en Afrique subsaharienne, les raisons pour lesquelles certaines mères adolescentes abandonnent l'école tandis que d’autres réussissent à poursuivre leurs études ne sont pas toutes connues. Dans cet article, nous avons étudié les caractéristiques comportementales et sociodémographiques des mères célibataires, associées à l'abandon scolaire dans un échantillon de près de 18.791 filles camerounaises, qui ont eu leur premier enfant pendant l'adolescence.

**Méthodes:**

Nous avons utilisé les données d'un registre national des mères célibataires, collectées au cours des années 2005-2008 et 2010-2011. Une analyse bivariée et des modèles de régression binaire logistique ont été utilisés pour explorer la relation entre la maternité à l'adolescence et l’abandon scolaire, en contrôlant une série de caractéristiques socio-économiques, familiales, sexuelles et sanitaires.

**Résultats:**

Parmi les 18 791 mères célibataires, 41,6% avaient abandonné l'école en raison d'une grossesse. Le modèle de régression multivariable a montré que l'abandon scolaire était plus fréquent chez celles qui avaient été expulsées du domicile parental (ORa : 1,85 ; IC 95% : 1,69 -2,04), chez celles qui déclaraient avoir d'autres mères célibataires dans leur famille (ORa : 1,16 ; IC 95% 1,08-1,25) et chez les mères qui avaient eu leur premier enfant avant 15 ans. L'utilisation de méthodes contraceptives modernes, le fait de n'avoir déclaré aucun partenaire sexuel au cours de la dernière année et d'avoir moins de deux enfants étaient associés à une probabilité réduite d'abandon scolaire.

**Conclusions:**

Un soutien social fort est essentiel pour assurer la continuité scolaire dans cette population vulnérable. L'abandon de l'école peut exposer la mère adolescente à un risque accru de comportements sanitaires dangereux et de nouvelles grossesses.

## Background

Despite all the policies and interventions that have been implemented throughout the world to prevent teenage pregnancies, they continue to be a challenge, especially in developing countries. According to the United Nations Population Fund, approximately 12 million girls aged 15 to 19 years and at least 777,000 girls under 15 years give birth each year in developing regions [1]. While the adolescent birth rate (ABR) that is, the number of births per 1000 women aged 15 to 19, has fallen in most countries, it is still rising in low-income settings. In sub-Saharan Africa, the ABR rose from 98 between 2010 and 2015, to 104 between 2015 and 2020 [2, 3]. According to the World Health Organization (WHO), adolescence is the period of life between 10 and 19 years old [4].

Research has shown that teenage pregnancies increases the risk of adverse health and social consequences for both the mother [5–7] and her child [8–11]. Children of teenage mothers are much more likely to have lower health assessment scores, lower academic achievement and higher risk of not completing high school during adolescence, compared to those of women that have their first birth at age 20 to 21 [12].

There is compelling, worldwide evidence that pregnancy and birth during teenage years are significant contributors to high school dropout rates [13–22]. A population-based study carried out in Cameroon revealed that pregnancy is the second cause of school dropout in girls with 16.9% dropout rates, after financial problems [23]. School dropout is therefore an additional barrier these adolescent girls who are already severely handicapped by parenthood, must face to overcome the longer-term repercussions of childbearing. Some studies have argued that the long-term negative consequences of early birth on a mother’s social status and child development could be lessened by continuing education, after an adolescent birth, suggesting that teenage single mothers' access to education should be strongly encouraged. Educational continuity may reduce the risk of subsequent pregnancies, enable teenage mothers to access more lucrative jobs and ensure improved cognitive and behavioral outcomes among children [9, 24–26]. However, the reasons why some teenage mothers drop out of school and others do not, are still unclear especially in sub-Saharan African settings.

Given the importance of education in girls, we aimed to investigate the background and behavioral characteristics of single mothers, associated with school dropout in a sample of 18,791 Cameroonian girls, who had their first child during adolescence. The purpose of this study is to drive the development of strategies to prevent school dropout and promote continuing education within this vulnerable population.

## Methods

### Study population

We used data from a national registry of single mothers who had their first child during adolescence, involved in the RENATA (Réseau National des Tantines) project. RENATA is the French acronym for the National Network of Aunties Associations, an aunty being a teenage mother trained in sexual and reproductive health and rights of adolescents.

This network consists of more than 20,000 single mothers trained as “Aunties” in about 305 community-based associations in Cameroon. RENATA is supported by a multidisciplinary team of psychologists, anthropologists and communication specialists. The recording of the profile of teenage mothers began in 2003 and this data was collected before each training session of a new Aunties' association in the community and continued systematically until 2012, nationwide. The main aim of the RENATA network is to ensure psychosocial support to single mothers through awareness about sexual abuse, violence against girls and harmful practices such as breast ironing and other forms of injustice against the girl child. The network’s activities also involve counseling in sexual and reproductive health, promotion and support of initiatives to empower and protect teenage mothers. The RENATA network received initial funding from the German cooperation through the German-Cameroon Health Programme implemented by the Deutsche Gesellschaft für Internationale Zusammenarbeit (GIZ) [27].

### Sampling technique and sample size

This is a retrospective review of the RENATA’s network database. Every girl who gave birth to one or more children between 10 and 19 years and was not married at the time of birth of the child was considered a teenage mother.

The inclusion criteria were: having given birth to at least one child between 10 and 19 years, being single and younger than 30 years at the time of the survey, 30-year-old was the age limit to be eligible to be part of the network.

Ten surveys were conducted between 2003 and 2012 and 23,723 adolescent mothers included. For this review, we excluded data collected before the year 2005 which did not include information on the participants’ reported sexual behavior like: the number of sexual partners during the last 12 months, history of rape, use of condom during the last sexual intercourse and use of a modern contraceptive method. Data from the years 2009 and 2012 were also excluded because of the small number of individuals included those years. With these restrictions, the resulting sample that was used for analyses was 18,791 single mothers recruited between 2005–2008 and 2010–2011.

### Dependent variable

The dependent variable was school dropout due to unplanned pregnancy or childbirth.

### Independent variables

The independent variables were the socioeconomic characteristics of the single mother: age at 1st childbirth in 2 groups (10–14 years and 15–19 years), the current age of the single mother, her educational level (vocational school, primary, secondary and higher), the number of children she has, the total amount of expenses during the last month and the region of residence (sahel, forest, coastal and grassfield region) that refers to the socio-anthropological areas of the country in which she lives. Another set of independent variables was the family background. The participants were asked if they have been sent away from their family house because of the pregnancy, if there were other cases of unplanned/early pregnancies within their family, what was the profession of the father of their last child and if the father was taking care of the child. The reported sexual and health seeking behavior of the teenage mothers were: age at 1st sexual intercourse, number of sexual partners during the last 12 months, history of rape, history of induced abortion, HIV screening and the use of modern contraceptive methods. These variables were assessed, based on participants’ self-reports.

### Statistical analyses

We used univariate analyses to describe the teenage mother’s population (proportions, percentiles, means ± standard deviation (SD)). Bivariate analyses were conducted to compare single mother school dropouts to non-school dropouts socioeconomic, sexual behavior and family background (Student t tests and *Χ*^2^ tests).

A multivariable binary logistic regression was conducted using the stepwise forward modelling to estimate the odds ratio (OR) of school dropout in teenage mothers. The model was adjusted for the year of inclusion in the study to address potential effect of the year of inclusion, as our preliminary analyses found a statistically significant correlation between school dropout and the year of registration to the network. The model included all control variables simultaneously, except the current age of the teenage mother. It allowed estimating the likelihood of school dropout according to the different factors associated to school dropout. Odd Ratios (OR) and adjusted ORs with their 95% confidence intervals (95% CI) were computed, and all indicators were estimated using the statistical software SPSS® version 16 (SPSS, Chicago, IL).

## Results

Among the 18,791 single mothers, 42.3% had dropped out of school because of unplanned pregnancy. The main demographic, family and sexual behavioral characteristics of the single mothers are presented in Table 1. The mean ± SD age at the time of the study was 20.5 ± 2.9 years. Overall, 33.4% of the mothers declared having at least two children. Over 60% of the single mothers had completed at least secondary school with only 0.8% at university at the time of the baseline survey. More than half (51.3%) of the mothers admitted that they had lost years of schooling. The mean age at 1st birth ± SD was 17.2 ± 1.5 in non-dropouts, compared to that of school dropout teenage mothers (16.4 ± 1.6; P < 0.001). Among the dropouts, 16.3% declared having been sent away from the family house because of pregnancy. Over half of the single mothers (57.3%) declared that the father of the most recent child was taking care of his/her needs. More than two-thirds (69.0%) of the participants reported having other family members who gave birth during adolescence. This proportion was higher in dropouts than in non-dropouts (71.5% *vs.* 67.2%; P < 0.000). The median amount (25th–75th percentiles) of expenses of the participants during the last month was 30 (20–60 euros).

**Table 1:** Association between sociodemographic, family and reproductive characteristics, and school dropout among single mothers in Cameroun

Variables	Total (N = 18,791)	Dropouts (n = 7941)	Non-dropouts (n = 10,850)	p-value
*Sociodemographic characteristics*				
Mean ± SD age at the time of the study (years)	20.5 ± 3.0	20.4 ± 3.0	20.6 ± 2.9	0.001
Mean ± SD age at 1st child’s birth of the (years)	17.1 ± 1.5	16.7 ± 1.6	17.1 ± 1.5	0.001
Age at 1st child’s birth (%)				
10–14	1244 (6.6)	654 (8.2)	590 (5.4)	0.001
15–19	17,547 (93.4)	7287 (91.8)	10,260 (94.6)	
Level of education (%)				
Vocational school	395 (2.1)	99 (1.2)	296 (2.7)	0.001
Primary school	6850 (36.5)	2021 (25.5)	4829 (44.5)	
High school/University	11,546 (61.4)	5821 (73.3)	5725 (52.8)	
Socioantropological region (%)				0.001
Coast	4395 (23.4)	1553 (19.6)	2842 (26.2)	
Forest	9188 (48.9)	4670 (58.8)	4518 (41.6)	
Grassfield	4565 (24.3)	1498 (18.9)	3067 (28.3)	
Sahel	643 (3.4)	220 (2.8)	423 (3.9)	
Number of children (%)				0.001
1	12,528 (66.7)	5014 (63.1)	7514 (69.3)	
2–3	5722 (30.5)	2694 (33.9)	3028 (27.9)	
≥ 4	541 (2.9)	233 (2.9)	308 (2.8)	
Amount of expenses during last month (%)				
< 20 USD	6455 (34.4)	2565 (32.3)	3890 (35.9)	0.001
20–50 USD	6380 (34.0)	2687 (33.8)	3693 (34.0)	
≥ 50 USD	5956 (31.7)	2689 (33.9)	3267 (30.1)	
*Family environment*				
Sent away from family house (%)				0.001
Yes	2317 (12.3)	1298 (16.3)	1019 (9.4)	
No	16,474 (87.7)	6643 (83.7)	9831 (90.6)	
Father providing for the child needs (%)				0034
Yes	10,765 (57.3)	4478 (56.4)	6287 (57.9)	
No	8026 (42.7)	3463 (43.6)	4563 (42.1)	
Family history of teenage mothers (%)				0.01
Yes	12,970 (69.0)	5678 (71.5)	7292 (67.2)	
No	5821 (31.0)	2263 (28.5)	3558 (32.8)	
Partner’s profession (%)				0.001
Student	5018 (26.7)	2527 (31.8)	2491 (23.0)	
Trader	2551 (13.6)	926 (11.7)	1625 (15.0)	
Craftsmen	7623 (40.6)	2929 (36.9)	4694 (43.3)	
Other	801 (4.3)	354 (4.5)	447 (4.1)	
Unemployed	1787 (9.5)	770 (9.7)	1017 (9.4)	
Civil servant	1011 (5.4)	435 (5.5)	576 (5.3)	
*Reported sexual behaviour*				
Mean ± SD age at 1st sexual intercourse (years)	15.4 ± 1.7	15.2 ± 1.7	15.4 ± 1.7	0.001
Number of sexual partners during the last 12 months (%)				0.001
0	1092 (5.8)	429 (5.4)	663 (6.1)	
1	13,390 (71.3)	5456 (68.7)	7934 (73.1)	
≥ 2	4309 (22.9)	2056 (25.9)	2253 (20.8)	
Was raped (%)				
Yes	1418 (7.5)	658 (8.3)	760 (7.0)	0.001
No	17,373 (92.5)	7283 (91.7)	10,090 (93.0)	
Ever had an induced abortion (%)				
Yes	3623 (19.3)	1542 (19.4)	2081 (19.2)	0.682
No	15,168 (80.7)	6399 (80.6)	8769 (80.8)	
Ever done HIV screening test (%)				
Yes	12,229 (65.1)	5087 (64.1)	7142 (62.8)	0.012
No	6562 (34.9)	2854 (35.9)	3708 (34.2)	
Use of a modern contraceptive method (%)				
Yes	7334 (39.0)	3133 (39.5)	4201 (38.7)	0.311
No	11,457 (61.0)	4808 (60.5)	6649 (61.3)	
Use condom during the last sexual intercourse (%)				
Yes	6162 (32.8)	2698 (34.0)	3464 (31.9)	0.003
No	12,629 (67.2)	5243 (66.0)	7386 (68.1)	
Ever had and STD (%)				
Yes	3760 (20.0)	1325 (16.7)	2435 (22.3)	0.001
No	15,031 (80.0)	6616 (83.3)	8415 (77.7)	
Had an ANC visit during pregnancy (%)				
Yes	17,900 (95.3)	7571 (95.3)	10,329 (95.2)	0.65
No	891 (4.7)	370 (4.7)	521 (4.8)	

The mean ± SD age at first sexual intercourse was 15.4 ± 1.7 years old. Almost one in five (19.3%) said they had an induced abortion at least once. HIV screening during the last year was performed by only 65.1% of the mothers and only 32.8% declared having used condom during their last sexual intercourse. Participants who reported more than one sexual partner during the last 12 months and those who declared having ever being raped were more likely to drop out from school (8.3% vs. 7.0%; P < 0.001).

Results of the multivariable model are presented in Table 2. School dropout was positively associated with being sent away from the parental home because of pregnancy (aOR: 1.85; 95% CI: 1.69–2.04). Single mothers who declared a primary or vocational school education level at the time of the study, had lower likelihood of SDO than those with high school or University education level (aOR: 0.40; 95% CI 0.37–0.42 and aOR: 0.30; 95% CI 0.23–0.38 respectively). Those who declared having single mothers in their family were more likely to dropout. Compared to those who had their first child after 14 years old, mothers who had their first child before had a 50% increased likelihood of school dropout. School dropout was more likely found in participants whose last child’s father was not providing for (aOR: 1.20; 95% CI 1.13–1.28) and in those who reported having never done an HIV screening test (aOR: 1.15; 95% CI 1.08–1.23). The likelihood of SDO increased with the number of partners or the number of children.

**Table 2:** Factors associated to school dropout in 18,791 teenage single mothers recruited between 2005 and 2011, in Cameroon

	n	OR	95% C.I	p-value	OR^a^	95% C.I	p-value
Age at first childbirth							
10–14 ans	1244	1.11	0.99–1.24	0.07	1.49	1.32–1.69	0.001
15–19 ans	17,547	1			1		
Educational level							
Vocational school	395	0.33	0.27–0.42	0.001	0.30	0.23–0.38	0.001
Primary school	6850	0.42	0.40–0.44	0.001	0.40	0.37–0.42	0.001
High school or University	11,546	1			1		
Sent away from home							
Yes	2317	1.27	1.17–1.38	0.001	1.85	1.69–2.04	0.001
No	16,474	1			1		
Father providing for the child needs							
Yes	10,765	1			1		
No	8026	1.41	1.35–1.47	0.001	1.20	1.13–1.28	0.001
Family history of teenage mothers							
Yes	12,970	1.28	1.23–1.33	0.001	1.16	1.08–1.25	0.001
No	5821	1			1		
Number of sexual partners during the last 12 months							
0	1226	0.65	0.57–0.73	0.001	0.79	0.68–0.91	0.001
1	14 758	0.69	0.66–0.71	0.001	0.86	0.80–0.92	0.001
≥ 2	4713	1			1		
Use condom during the last sexual intercourse							
Yes	6162	1			1		
No	12,629	1.28	1.22–1.35	0.001	1.01	0.94–1.07	0.873
Ever done HIV screening test							
Yes	12,229	1			1		
No	6562	1.41	1.22–1.35	0.001	1.15	1.08–1.23	0.001
Number of children							
1	12,528	0.67	0.64–0.69	0.001	0.72	0.60–0.87	0.007
2–3	5722	0.89	0.84–0.94	0.001	1.03	0.85–1.25	0.409
≥ 4	541	1			1		
Was raped							
Yes		1			1		
No		0.87	0.78–0.96	0.007	0.88	0.78–0.98	0.026

^a^Adjusted for every variable presented and year of inclusion in the study, socioanthropological region, partner’s profession, amount of expenses during the last month, condom use during the last sexual intercourse, age at first sexual intercourse, ever had an STD

## Discussion

The results of this data review on the largest network of single in Cameroon showed that more than half of the girls (54%) had to drop out of school for at least some time because of pregnancy and childbirth, and 77% of them (46.1% of all mothers) dropped out of school permanently. Early age (less than 14 years) at 1st childbirth and factors like having being sent away from home because of pregnancy, the father’s child not providing for him/her, existence of other single teenage mothers in the family and history of rape were associated with increase odds of school dropout, while responsible personal health behaviors such as having only one or no sexual partner during the previous year, the use of modern contraceptive methods and voluntary HIV testing were associated with decreased odds of school dropout. These associations remained statistically significant after controlling for potential confounding factors related to the teenage sociodemographic, family and sexual behavioral characteristics.

The rate of school dropout in this study population is higher than that of girls in the national sample of the Multiple Indicators Cluster Survey in Cameroon (16%) [28]. This is understandable given the fact that pregnancy and childbearing are among the main determinants of school dropout in teenage adolescents and our study population is a selection of teenage mothers [29]. Indeed, several studies have highlighted the high prevalence of SDO among teenage mothers [13, 22, 30–34]. Timæus et al. prospectively demonstrated that relationship in a cohort of 673 childless and school teenage girls. Girls who subsequently gave birth had 4.1 times the odds of other girls of dropping out of school [22].

Social and economic vulnerability of teenage mothers play a role in their ability to continue school or not. In this sample, mothers without family support neither from their parents because they were sent away from home nor from the child’s father, were more likely of dropping out of school. Similar conclusions were drawn by other authors who showed that, adolescent mothers living with their parents were more likely to stay in school or get back to school promptly after giving birth [22, 32]. This situation is compounded by the discrimination the girls may face from the community and particularly from their peers and teachers in school. Although several African countries have adopted laws and policies that protect the rights of adolescent girls to stay in school during pregnancy and motherhood, some continue to promote discriminatory and punitive measures against adolescent girls who become pregnant [35]. Pregnant adolescent girls are sometimes stigmatized, booed by their peers and teachers, or even excluded from schools [29, 36]. Even in countries that support the educational continuity of adolescent mothers, few have support structures in schools and communities to assist adolescent mothers who choose to stay in school [35]. SDO thus seems inevitable when teenage mothers are left in the front line of caring for their babies, without affective and financial support from their families and community, to cope with the responsibilities of and adjustment to parenthood. Studies have shown that adequate support from family and partner and positive interactions within the social network were associated with good maternal behavior, life satisfaction in mothers, and school continuity in adolescent mothers [6, 25]. Being deprived of this support in a country where professional support specifically targeted at this vulnerable population is not developed leads to the isolation of the teenage mother, accentuates the precariousness of her economic condition and leaves her without any chance to continue her education. Social support needs to be strengthened around adolescent mothers to encourage educational continuity. In particular, governments must commit to combating discrimination in schools based on pregnancy, in policy and practice, to ensure that there are no legal barriers to keeping teenage mothers in school.

In addition, SDO was associated with a risky sexual and health behaviour. This result is consistent with that of many other studies which stated that school attendance may influence sexual network and increase health-seeking behaviour [37–40]. Indeed, in a context marked by the scarcity of opportunities for professional integration, teenage mothers who leave school are inevitably exposed to unwanted sexual advances from adult males, coercive sexual relations, unequal gender power relations, lack of comprehensive sexuality education, non-use of contraceptives and inappropriate forms of recreation [41–45]. They are excluded from conventional channels of information on responsible sexuality and reproductive health and have limited access to the basic health services that are usually offered to adolescents in school settings. They thereby miss out on the opportunity to be exposed to all these services that may enable them to ensure good health for themselves and their offspring. Overall, studies have highlighted the poor access of teenage mothers to sexual and reproductive health services [44, 45]. Youth-centered and friendly reproductive health services that can accommodate young people during non-working hours, i.e. before or after school, should be developed within communities and particularly within schools to improve young people's access to reproductive health information and services.

In this study, 7.5% of the single mothers declared having ever been raped and those ever been raped were more likely to drop out of school. This result is a further indicator of the vulnerability of single mothers and an indication of the urgency of scaling up interventions to raise awareness about sexual abuse, violence, and other harmful practices against children in general and specifically the girl child. The association between early age at first childbirth and SDO highlighted by this study suggests that adolescents and families should be exposed early enough to sexual education to postpone the age at first birth and reduce the risk of SDO. Given the increasing importance of female school participation in sub-Saharan Africa, sexual education should not be limited in schools or health facilities but should be an integrated part of the education given by parents in homes.

This study is the first to present the association between childbearing and school attendance in almost 19,000 single mothers living in a sub-Saharan country. Nevertheless, the study presents some limitations. The generalization of the results to all teenage mothers in the country must be cautious. This sample is mainly constituted of teenage mothers living in precarious socio-economic conditions, there may be differences in educational path among teenage mothers with different socio-economic background. However, the results obtained from this large-scale sample of 18,791 adolescent mothers from all regions of the country may likely reflect the characteristics of teenage mothers from disadvantaged backgrounds, in countries with socioeconomic and cultural characteristics like Cameroon’s. Additional limitations of this study included the difficulty controlling for any confounder related to the teenage parents’ household (single parents, number of siblings, sibling rank, etc.) because the information was not available in the database. Since this study is cross-sectional, no causal link can be established between school drop-out and the characteristics of adolescent mothers.

## Conclusions

The results of this study in 18,791 teenage mothers showed that strong social support is essential to ensure school continuity in this vulnerable population and dropping out of school may put the teenage mother more at risk of risky health behaviour and new pregnancy. While prevention of teenage pregnancy should continue to be a public health priority for governments, measures must be taken at all levels—individuals, communities and governmental—to ensure the continuity of education of adolescent mothers. Several studies have shown that the negative consequences of early childbirth for mothers and children can be mitigated by further education. Thus, social support programmes targeting adolescent mothers like childcare solutions or alternative education programmes must be developed and implemented by governments or communities, to support teenagers and families struggling to cope with early pregnancy and parenthood.

## Data Availability

The datasets used and/or analysed during the current study are available from the corresponding author on reasonable request.

## Abbreviations

ABR: Adolescent birth rate
AOR: Adjusted odd ratio
BMZ: German Federal Ministry of Cooperation
CI: Confidence interval
GIZ: Gesellschaft für Internationale Zusammenarbe
HIV: Human immunodeficiency virus
IQR: Interquartile range
OR: Odd ratio
PGCSS: German-Cameroon Health Programme
RENATA: Reseau National des Tantines
SDO: School dropout
UNFPA: United Nations Population Fund
WHO: World Health Organization
